# Characterization of Reagent Pencils for Deposition of Reagents onto Paper-Based Microfluidic Devices

**DOI:** 10.3390/mi8080242

**Published:** 2017-08-05

**Authors:** Cheyenne H. Liu, Isabelle C. Noxon, Leah E. Cuellar, Amanda L. Thraen, Chad E. Immoos, Andres W. Martinez, Philip J. Costanzo

**Affiliations:** Department of Chemistry and Biochemistry, California Polytechnic State University, San Luis Obispo, CA 93401, USA; cliu16@calpoly.edu (C.H.L.); inoxon@calpoly.edu (I.C.N.); leahcuellar.chem@gmail.com (L.E.C.); athraen@calpoly.edu (A.L.T.); cimmoos@calpoly.edu (C.E.I.)

**Keywords:** paper, diagnostics, microfluidic paper-based analytical devices (microPADs), pencil, wear, colorimetry

## Abstract

Reagent pencils allow for solvent-free deposition of reagents onto paper-based microfluidic devices. The pencils are portable, easy to use, extend the shelf-life of reagents, and offer a platform for customizing diagnostic devices at the point of care. In this work, reagent pencils were characterized by measuring the wear resistance of pencil cores made from polyethylene glycols (PEGs) with different molecular weights and incorporating various concentrations of three different reagents using a standard pin abrasion test, as well as by measuring the efficiency of reagent delivery from the pencils to the test zones of paper-based microfluidic devices using absorption spectroscopy and digital image colorimetry. The molecular weight of the PEG, concentration of the reagent, and the molecular weight of the reagent were all found to have an inverse correlation with the wear of the pencil cores, but the amount of reagent delivered to the test zone of a device correlated most strongly with the concentration of the reagent in the pencil core. Up to 49% of the total reagent deposited on a device with a pencil was released into the test zone, compared to 58% for reagents deposited from a solution. The results suggest that reagent pencils can be prepared for a variety of reagents using PEGs with molecular weights in the range of 2000 to 6000 g/mol.

## 1. Introduction

Paper-based diagnostic tests comprise two essential components: the paper-based platform (i.e., the device) and the reagents for the assay. The reagents are most commonly deposited on the device by preparing a solution of the reagent in an appropriate solvent, depositing a volume of the reagent solution on the device, and then drying the device to remove the solvent, thus leaving the dry reagent on the device. Our research groups introduced an alternative approach for depositing reagents in a solvent-free process using reagent pencils [1]. Reagent pencils are solid cores made by pressing a mixture of polyethylene glycol (PEG), graphite, and the desired reagent into the shape of a pellet. The pencil can then be used to deposit reagents on a device by simply drawing on the device to create a pencil trace. Once a sample solution is introduced into the device, it dissolves the reagent from the pencil trace so that it becomes available to react with the analyte and perform an assay. Our initial work with reagent pencils demonstrated that they could be used to deposit reagents on paper-based microfluidic devices for performing a glucose assay, and that the shelf-life of enzymes was extended significantly in the pencil. In this article, we describe our work on characterizing the pencils in more detail to elucidate the relationships between the composition of the pencil core, the amount of material deposited on a device, and the amount of reagent that dissolves into a sample and is available for performing an assay.

Paper-based diagnostic tests have been developed extensively for applications in both clinical and analytical chemistry because of their accessibility, versatility, and simplicity [2,3,4]. The need for paper-based diagnostic tests stems from the demand for simple tests that can be performed on-site or at the point of care with minimal supporting infrastructure. Successful paper-based diagnostics also have the potential to create a positive impact in both personal and global healthcare by increasing access to diagnostics, reducing cost, and improving patient compliance [2]. Several types of paper-based diagnostics have been developed, including paper chromatography [5], dipstick assays [6], and lateral-flow immunoassays [7,8,9]. Paper-based tests typically provide qualitative results on their own, but, when combined with an instrument such as a smartphone or an electrochemical reader, they can also be used to perform quantitative tests [2].

Microfluidic paper-based analytic devices (microPADs) are a relatively new class of paper-based devices that combine features from microfluidic, lateral flow, and dipstick devices [2,10,11]. Traditional microfluidic devices are made by etching or molding channels into glass, silicon, or poly(dimethylsiloxane) (PDMS) and typically require pumps to move fluids through the device [12,13]. MicroPADs, on the other hand, are made by patterning hydrophilic porous membranes (e.g., paper) with hydrophobic barriers to create networks of hydrophilic channels and test zones in which aqueous samples can wick via capillary action [14]. Diagnostic assays are conducted on microPADs by wicking samples into different test zones where the analyte can react with pre-deposited reagents. These devices effectively eliminate the need for an active pumping component generally associated with traditional microfluidic devices and are much cheaper and easier to fabricate than conventional microfluidic devices [10]. A recent surge of research focused on the development of microPADs has established their versatility as a platform for conducting both qualitative and quantitative assays [2,3,10]. The combination of microPADs and reagent pencils presents opportunities for developing customizable tests at the point of care.

Pencils are simple, portable, low-cost writing tools that have been developed for various applications beyond scripture [1,15,16]. A pencil works by abrading material from the pencil core onto a given surface as the tip of the pencil is dragged across the surface [17], and pencils provide a convenient method for depositing small amounts of material on paper in a solvent-free process [1]. Reagent pencils harness this capability and were developed for storing and depositing reagents onto microPADs. Although microPADs generally only require microgram quantities of reagents to perform an assay, the reagents must be stable for long periods of time at room temperature in order to be shipped for use in remote settings [2]. Regent pencils offer the opportunity to deposit reagents on devices at the point of care, so that the reagents can be stored safely for extended periods of time in the pencil core and then be deposited freshly on the device immediately before performing an assay [1]. To make reagent pencils, a particular reagent or set of reagents is combined at concentrations up to 15% *w*/*w* with a mixture of 75% polyethylene glycol and 25% graphite by mass, and pressed into a cylindrical pellet that we call the pencil core. The pencil cores can then be loaded into mechanical pencil holders to facilitate the deposition of reagents onto microPADs. Our initial work with reagent pencils was focused on developing a method for fabricating reagent pencils and on demonstrating some of the most significant applications and attributes of these tools. We have now performed a more detailed characterization of reagent pencils in terms of their wear resistance, which is a function of the total mass of material deposited on a device, and the efficiency of pencil cores for delivering reagents to the test zones of microPADs.

To characterize reagent pencils in more detail, this work examined the effects of pencil core diameter, polymer composition, reagent type, and reagent concentration on the wear resistance (i.e., the amount of reagent pencil deposited on a device during a trace), reagent release characteristics (i.e., how the reagent distributes itself on the device when an aqueous sample comes in contact with a pencil trace), and reagent release efficiencies (i.e., the percentage of total reagent deposited on the device that becomes available to participate in the chemical reaction linked to a diagnostic assay) of reagent pencils. To determine wear resistance, a test for pin abrasion was used [18]. To determine reagent release characteristics, Erioglaucine, a water-soluble, small molecule blue dye was used to monitor the distribution of the reagent on the device colorimetrically [1]. To determine reagent release efficiencies, Erioglaucine was also used as a model analyte, but it was extracted from the devices into a solution and then measured via absorption spectroscopy.

## 2. Materials and Methods

All reagents and materials were purchased from commercial sources unless stated otherwise. The following reagents, materials, and equipment were used: poly(ethylene glycol) (PEG, Mn ~600, 2000, 3400, 6000 g/mol, Sigma Aldrich, St. Louis, MO, USA), poly (ethylene glycol) dimethyl ether (PEGdiME, Mn ~2000, Sigma Aldrich), graphite powder (General’s Pure Powdered Graphite), Erioglaucine disodium salt (blue dye, Alfa Aesar, Haverhill, MA, USA), dextrose (glucose, Sigma Aldrich), horseradish peroxidase (HRP, 67 U/mg, MP Biomedicals, Santa Ana, CA, USA), glucose oxidase (GOx, 266 U/mg, MP Biomedicals), 2,2′-azino-bis(3-ethylbenzothioazoline-6-sulfonic acid) diammonium salt (ABTS, Alfa Aesar), 1× phosphate-buffered saline (1XPBS pH 7.4, prepared from a 10× solution, Thermo Fisher Scientific, Waltham, MA, USA), chromatography paper (Whatman No. 1 CHR, GE Healthcare Life Sciences, GE Healthcare, Chicago, IL, USA), manual pellet press equipped with a 0.25 inch (6.35 mm) or a 0.125 inch (3.18 mm) diameter punch and dye set (Parr Instrument Company, Moline, IL, USA), analytical balance (Sartorius AG, Goettingen, Germany), flatbed scanner (Epson Perfection V300, Epson, Nagano, Japan), plotting cutter (Graphtec Plotting Cutter CE6000-40, Graphtec America, Irvine, CA, USA), 6.0-mm mechanical pencil holders (Art Alternatives, San Francisco, CA, USA), 3.2-mm mechanical pencil holders (Faber-Castell, Stein, Germany), solid ink printer (Phaser 8560, Xerox, Norwalk, CT, USA), convection oven (MTI corporation, Richmond, CA, USA), rotary evaporator (Buchi, BÜCHI Labortechnik AG, Flawil, Switzerland), lyophilizer (Labconco, Kansas, MO, USA), micropipets (Gilson, Middleton, WI, USA), and a spectrophotometer (Cary 100 UV-vis, Agilent Technologies, Santa Clara, CA, USA).

Reagent pencils were fabricated using a modified version of the original method [1]. For each reagent pencil, a mixture of 75% polymer (PEG or PEGdiME) and 25% graphite powder by mass was prepared. For pencils containing mixtures of polymers, the polymers were mixed in the given ratio by mass and then a mixture of 75% polymer mixture and 25% graphite by mass was prepared. Reagents (Erioglaucine, glucose, and HRP) were then added to the polymer/graphite mixture at various concentrations ranging from 0 to 15% *w*/*w*. A slurry of the resulting polymer/graphite/reagent mixture was prepared in either acetone or nanopure water. Slurries prepared in acetone were rotary evaporated at 80 °C to remove the solvent. Slurries prepared in water were frozen in liquid nitrogen and then lyophilized for 24 h. The resulting dry mixtures were then pressed manually using a pellet press with a force of approximately 45 kN to create cylindrical pellets with diameters of 6.35 or 3.18 mm. These pellets were loaded into mechanical pencil holders to facilitate the reagent deposition onto microPADs. The pencil cores were stored in a desiccator at room temperature.

MicroPADs were fabricated via wax printing [19]. The devices were designed using AutoCAD and printed on chromatography paper using a solid-ink printer. Two devices were used for this project, a bone device and a caterpillar device, which are shown in Figures 2 and 6, respectively. The bone device had a sample addition zone, a channel, and a test zone, and all three segments were designed to have the same surface area. The caterpillar device had a sample addition zone, five circular reagent deposition zones, and a test zone, all connected by a channel. After printing the designs on the paper, the paper was placed in a convection oven set to 145 °C for 15 min. The devices were allowed to cool to room temperature under ambient conditions and were stored wrapped in aluminum foil until they were used.

A wear resistance test was performed to characterize the wear of the pencil cores. The pencil core was placed in a mechanical pencil holder and loaded into a cutting plotter in place of a blade. The plotter was then used to trace the pencil across a piece of chromatography paper in straight, parallel lines for a total distance of 8.17 m. The plotter ensured that a constant contact area, force, velocity, and distance traced was achieved for each trial. The change in mass of the pencil core for each trial was recorded using an analytical balance, and the wear resistance was calculated as adapted from ASTM G132-96 [19]. Wear was calculated using the following equation:

(1) $$wear=\;\frac{CW_{x}}{\rho S_{x}}\;{\mathrm{mm}}^{3}/\mathrm{Nm}$$

where *W_x_* is the mass loss of the pencil core, *S_x_* is the mass loss of a reference pencil core (a reagent pencil made with 75% PEG 2000 and 25% graphite powder by mass), *ρ* is the density of the core (g/cm^3^), and *C* is a reference constant (mg/mN). The magnitude of the reference constant, *C*, was calculated as the mean mass loss (mg) of the reference sample per unit track length (m) per unit load (N). For all samples, the unit track length was 8.17 m, and the load was 2.94 N [20]. The reference reagent pencil under these conditions was found to have a mean mass loss of 12.97 mg.

Erioglaucine disodium salt was used as a model analyte to study the delivery of reagents from a pencil trace to the test zone of a device. Pencil cores containing Erioglaucine were used to manually deposit the reagent in the sample zone of bone devices. The pencil was traced across the sample zone in vertical, adjacent, and parallel lines to cover the zone completely while avoiding any overlap between adjacent pencil traces. An aliquot of nanopure water (13 µL) was then added to the sample zone of the device (on top of the pencil traces) using a micropipette. The water dissolved the Erioglaucine from the pencil traces and transported it into the test zone. The devices were allowed to dry under ambient conditions for 30 min and then the mean intensity of the color in the test zone was determined via digital image colorimetry (DIC) [21]. First, the devices were scanned, then the image was analyzed in ImageJ 1.46r by inverting the image, splitting it into the red, green, and blue color channels, and, finally, selecting the entire circular area of the test zone in the red channel and recording the mean intensity. The background signal was obtained by performing the same procedure but using a pencil core containing no Erioglaucine.

**Figure A1:**
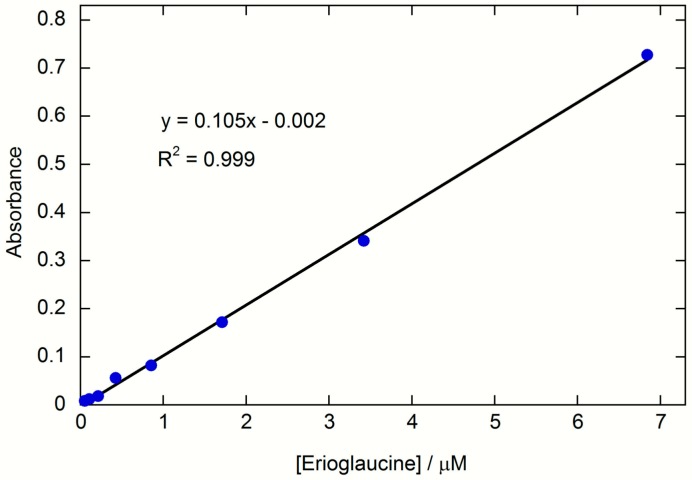
External calibration curve prepared using standard Erioglaucine solutions that was used to determine the quantity of Erioglaucine extracted from the devices for the efficiency of the reagent delivery experiments. The data was fit with a linear trendline in Kaleidagraph.

To determine the efficiency of the delivery of reagents to the test zone, reagent pencils containing 5% *w*/*w* Erioglaucine were used to manually deposit the reagent on the sample zone, channel or test zone of a bone device. For the sample zone and test zone, the reagents were deposited as described previously. For the channel, the pencil was traced across the channel in a single horizontal trace. Nanopure water (13 µL) was then added to the sample zone and allowed to wick into the test zone. Once the device was dry, it was scanned and the color intensity of the test zone was determined via DIC. Then, the sample zone, channel, and test zone were cut apart with scissors, placed in a microcentrifuge tube containing 1.00 mL of nanopure water, and the Erioglaucine was extracted for 1 h with shaking. The absorbance of the resulting solutions was measured and compared to a calibration curve prepared using standard Erioglaucine solutions with concentrations of 6.84, 3.42, 1.71, 0.855, 0.427, 0.214, 0.107, and 0.053 µM (Figure A1). A series of control experiments were also performed where the Erioglaucine was deposited from a solution (1 µL of 2.74-mM Erioglaucine deposited in the sample zone, channel or test zone) instead of from a reagent pencil.

The final set of experiments was performed with the caterpillar device to illustrate the possibility of preparing calibration curves using a single reagent pencil. First, a reagent pencil containing 5% *w*/*w* Erioglaucine was used to manually deposit the reagent in varying numbers of the reagent deposition zones from 1 to 10—the caterpillar device has five reagent deposition zones, but the reagent pencil can be used to deposit reagent on the front and back face of each deposition zone, which gives a total of 10 deposition zones. Nanopure water (33 µL) was then added to the sample zone and allowed to wick into the test zone. After drying the devices under ambient conditions, the intensity of the color in the test zone was quantified via DIC. In a second experiment, devices were prepared for performing a glucose assay by depositing 1 µL of reagent solution (25 mM ABTS, 67 U/mL HRP, and 230 U/mL GOx prepared in 1× PBS) in the test zone and drying the devices under ambient conditions. A 5% *w*/*w* glucose pencil was then used to deposit glucose in one, two, three, four, or all five of the reagent deposition zones. An aliquot of 1× PBS (33 µL) was then added to the sample zone of the device and was allowed to wick into the test zone. After allowing the devices to dry for 30 min under ambient conditions, the intensity of the color in the test zone was measured via DIC.

## 3. Results and Discussion

**Figure 1:**
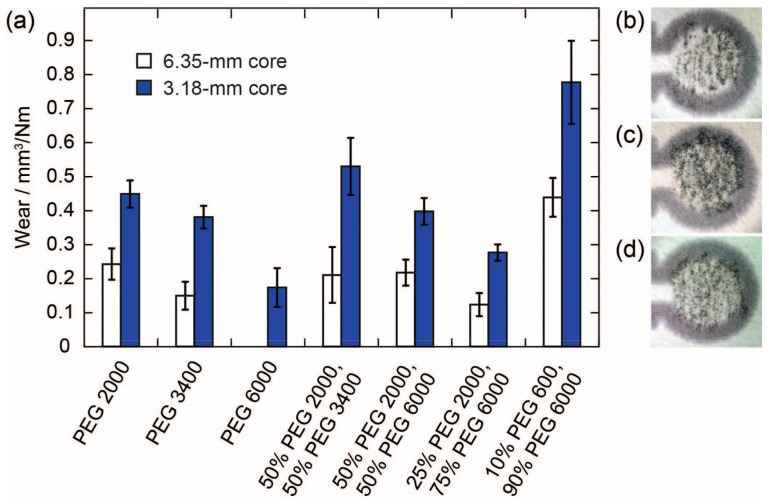
Effect of the pencil core diameter and molecular weight of PEG on the wear of reagent pencils: (**a**) Wear of pencil cores with two diameters (6.35 and 3.18 mm) fabricated using PEG with various molecular weights. The height of the bars represents the mean of six replicates and the error bars represent one standard deviation from the mean. Pencil cores made from PEG 6000 with a diameter of 6.35 mm did not hold together and crumbled during the experiment, so they were not included in this study; (**b**) Photograph of a pencil trace made with a PEG2 2000, 6.35-mm pencil core; (**c**) Photograph of a pencil trace made with a PEG 2000, 3.18-mm pencil core; (**d**) Photograph of a pencil trace made with a PEG 6000, 3.18-mm pencil core.

The magnitudes of the wear determined for pencil cores with two different diameters (6.35 and 3.18 mm) using PEGs of various molecular weights are shown in Figure 1. Overall, the wear for pencil cores with the smaller diameter was approximately twice as high as the wear for the cores with the larger diameter, for each respective PEG composition. We attribute this effect to a combination of two factors: first, since the same force was applied to both pencil cores, the smaller core was subject to a pressure four times larger than the larger core; and, second, the core with the smaller diameter was most likely able to make better contact with the surface of the paper—i.e., proportionally more surface area of the tip of the 3.18-mm core was able to make contact with the paper and deposit material on the paper. Images of pencil traces from large and small cores on paper confirm these results qualitatively (Figure 1b,c). The smaller pencil core was able to produce a darker and more uniform pencil trace on the paper. Based on this result, we conducted all further experiments described in this manuscript using the smaller, 3.18-mm-diameter pencil cores.

In addition to the diameter of the pencil core, the molecular weight (M_n_) of the PEG also has a significant effect on the wear of a pencil core (Figure 1a). We found that the higher the M_n_ of the PEG, the lower the wear of the pencil core. We attribute this effect to the length of the polymer chains, which in turn determines the strength of the intermolecular forces, the degree of polymer chain entanglement, and the number of polymer chain ends in the pencil cores. Polymers with higher molecular weights and longer polymer chains would be expected to have stronger intermolecular forces, more chain entanglements, and fewer chain ends compared to polymers with lower molecular weights. All three factors contribute to lower wear for the pencil cores made from the higher-molecular-weight polymers—stronger intermolecular forces and a higher degree of chain entanglement both lead to higher tensile strength and brittleness for a polymer; fewer chain ends reduces the free volume and limits the plasticization of a polymer. This effect is similar to what is observed for traditional pencils with various hardnesses. Harder pencils produce lighter traces on paper compared to softer pencils. The effect of the M_n_ of PEG on wear can also be seen qualitatively, as the core made from PEG 2000 produces a darker trace compared to the core made with PEG 6000 (Figure 1c,d). PEGs with M_n_ lower than 2000 g/mol produced cores that were too soft to be handled and loaded into the mechanical pencil holders. PEGs with M_n_ higher than 6000 g/mol could not be pressed into a pellet using the manual press. However, by combining PEGs with different molecular weights, it is possible to tune the wear of the pencil core; therefore, depending on the desired application, it should be possible to formulate a mixture of PEGs with the desired wear characteristics. The wear of a pencil core containing a mixture of PEGs was approximately equal to the weighted average of the wear of pencil cores made with the individual PEGs.

**Figure 2:**
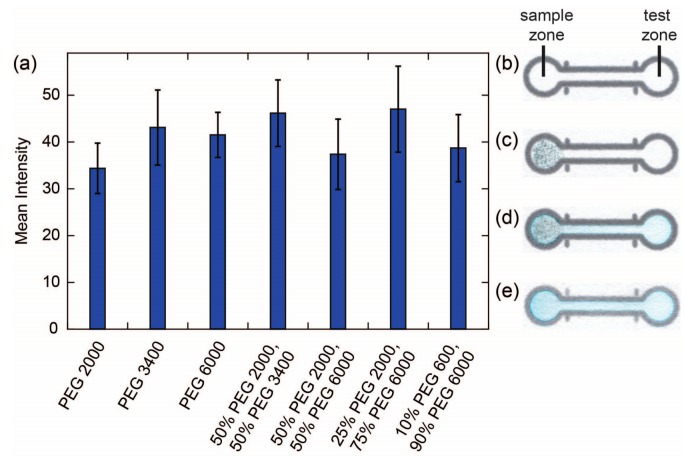
Delivery of Erioglaucine blue dye from a reagent pencil deposited in a sample zone to a test zone: (**a**) Mean intensity of the color measured in the test zone for pencils made from PEG with various molecular weights. The height of the bars represents the mean of 16 replicates and the error bars represent one standard deviation from the mean; (**b**) Device used to determine the delivery of Erioglaucine; (**c**) Device after coloring in the sample zone with a reagent pencil; (**d**) Device after adding water to the sample zone to dissolve the Erioglaucine from the pencil matrix and transport it into the test zone; (**e**) Bottom face of the device shown in (**d**) that was scanned and used for the analysis.

To determine whether there was a correlation between the wear of a pencil core and the amount of reagent delivered to a device, we prepared pencil cores using the same PEGs shown in Figure 1a with an additional 15% *w*/*w* Erioglaucine blue dye added so that we could monitor the reagent via DIC. We were somewhat surprised to find that all seven pencil cores delivered approximately the same amount of reagent to the test zone in this experiment (Figure 2). Two possible explanations for this observation were that the signal became saturated, so we were not able to see the differences between the amounts of Erioglaucine delivered to the test zones, or that the addition of the reagent to the pencil cores was affecting the wear of the pencil cores. The second explanation was supported by our observation that the pencil cores containing 15% *w*/*w* Erioglaucine appeared to be softer and tackier than the cores containing no added reagent.

**Figure 3:**
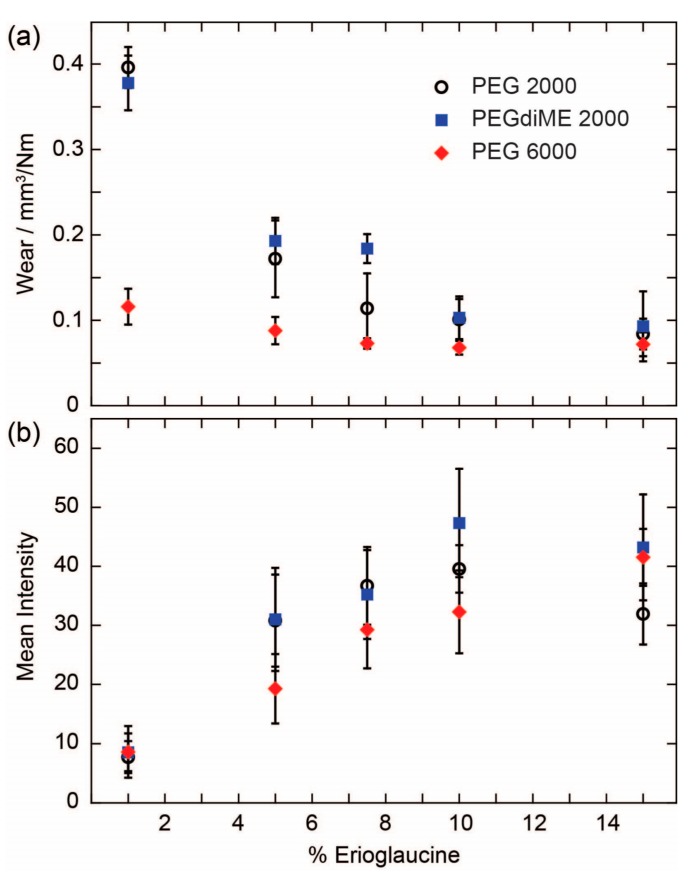
Effect of reagent concentration on the wear and delivery characteristics of reagent pencils: (**a**) Wear determined for pencil cores containing various concentrations of Erioglaucine and fabricated using three different PEGs. Data points represent the mean of six replicates and the error bars represent one standard deviation from the mean; (**b**) Mean intensity of the color measured in the test zones for the same pencil cores shown in (**a**). Data points represent the mean of 16 replicates and error bars represent one standard deviation from the mean.

To shed additional light on the results obtained with 15% Erioglaucine, we conducted a more detailed experiment where we prepared a series of pencil cores with varying concentrations of Erioglaucine and measured both their wear and the amount of reagent delivered to the test zone (Figure 3). The pencil cores for this experiment were prepared using PEG 2000, PEGdiME 2000, and PEG 6000. PEGdiME 2000 was tested in this experiment because it has the same molecular weight and approximately the same chain length as PEG 2000, but the chain ends are different, so it provided an opportunity to evaluate the effect of variations in polymer chain end composition on the performance of the pencil cores. Overall, the results show that the performance of reagent pencils is not affected significantly by slight modifications in the structure of the polymer chain ends. The results further show that the incorporation of Erioglaucine into the pencil core results in a significant decrease in wear in the cases of PEG 2000 and PEGdiME 2000. For PEG 6000, the wear also decreased upon incorporation of the Erioglaucine, but not as dramatically as for PEG 2000, presumably because the initial wear of the PEG 6000 pencil core was already low. At 15% *w*/*w* Erioglaucine, no statistically significant differences were observed between the wears of the three pencil cores (α = 0.05), which explains, at least in part, why no significant differences were observed between the results shown in Figure 2. The results from the reagent release experiment show a strong positive correlation between the concentration of reagent in the pencil core and the mean intensity of the signal measured from the test zone up to 10% *w*/*w* Erioglaucine for all three PEGs (Figure 3b). By comparing the results in Figure 3a,b, we can conclude that the amount of reagent released by a pencil trace is more strongly influenced by the concentration of reagent in the core than the wear of the core, at least up to 10% *w*/*w* reagent. Above 10% *w*/*w* Erioglaucine, the colorimetric signal appears to be saturated, which further explains the results shown in Figure 2. Finally, since the results in Figure 3 show that pencil cores made with PEGdiME 2000 have slightly higher wear and release slightly more Erioglaucine than cores made with PEG 2000 or PEG 6000, PEGdiME 2000 was selected as the polymer for fabricating all the remaining pencil cores used in this study.

**Figure 4:**
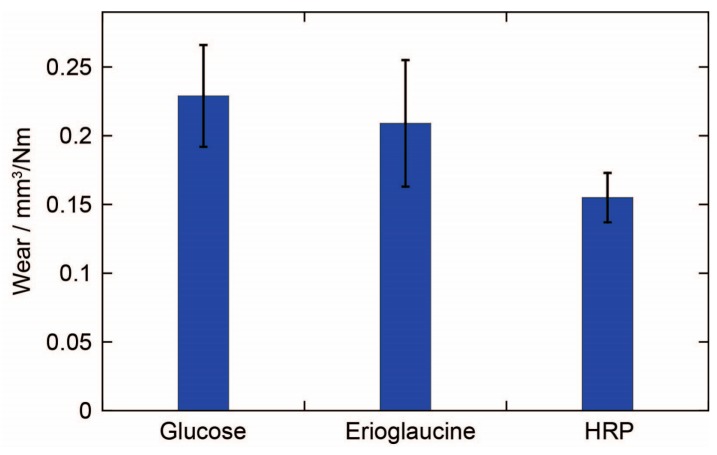
Effect of the reagent on the wear of reagent pencils made from PEGdiME 2000 via lyophilization. The reagents were all added to a concentration of 5% *w*/*w*. The height of the bars represents the mean of six replicates and the error bars represent one standard deviation from the mean.

The wear of a pencil core also appears to be influenced by the properties of the reagent itself (Figure 4). Glucose (molecular weight (m.w.) 180.2 g/mol), Erioglaucine (m.w 792.8 g/mol), and HRP (m.w. ~44,000 g/mol), all at a concentration of 5% *w*/*w*, produced pencil cores with different wear. The wear was found to have an inverse correlation with the molecular weight of the reagent. However, with such a small sample size of reagents, it is impossible to make a general conclusion about this relationship. More importantly, only a small difference in wear was observed between the pencil cores containing glucose and Erioglaucine, even though Erioglaucine has a molecular weight that is over four times larger than the molecular weight of glucose. So, the results suggest that the wear of a pencil core is more strongly influenced by the concentration of reagent rather than the molecular weight of the reagent, at least for small, water-soluble molecules.

**Figure 5:**
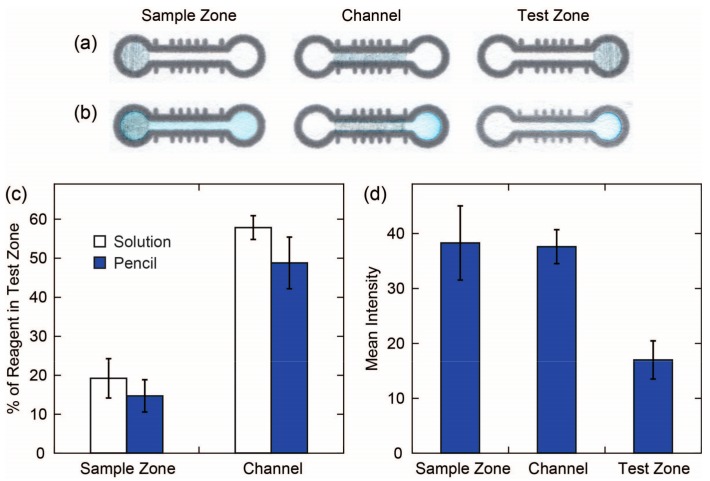
Efficiency of delivery of a reagent to a test zone: (**a**) Images of devices where reagents were deposited using a pencil in the sample zone, the channel and the test zone. All three regions had the same surface area so that the same amount of reagent was deposited in each case; (**b**) Same devices as in (**a**) after adding 13 µL of water to the sample zone. The back face of the device with reagent deposited in the test zone was imaged to avoid interference from the graphite in the pencil trace; (**c**) Bar graph of the percentage of the total amount of reagent deposited on a device that was transported into the test zone after the addition of water. The height of the bars represents the mean of seven replicates and the error bars represent one standard deviation from the mean; (**d**) Bar graph of the mean intensity of the color in the test zone obtained from the reagent deposited in the three zones of the device using a reagent pencil containing 5% *w*/*w* Erioglaucine. The height of the bars represents the mean of seven replicates and the error bars represent one standard deviation from the mean.

Our evaluation of the efficiency of the delivery of reagents deposited using reagent pencils showed that solution-based deposition is only slightly more efficient than pencil-based deposition of reagents in terms of the percentage of total reagent deposited on the device that ends up in the test zone and presumably is available to react during the assay (Figure 5 and Table A1). What was most surprising from these results was that the location where the reagent was deposited on the device, whether from solution or pencil, had such a strong influence on how much of the reagent ended up in the test zone. When the reagent was deposited in the sample zone, only ~15% of the reagent was transported into the test zone. The rest of the reagent remained in the sample zone and channel, as can be seen in the images in Figure 5b (first column). When the reagent was deposited in the channel, ~50% of the reagent was transported into the test zone. However, when looking at the signal from the test zone obtained via DIC, we see that the highest signal was obtained when the reagent was deposited in the sample zone. We attribute this effect to the distribution of the Erioglaucine in the test zone. When the reagent was deposited in the sample zone, a more uniform distribution of color was obtained in the test zone, which led to a higher colorimetric signal. When the reagent was deposited in the channel, even though more total reagent was transported into the test zone, a lower colorimetric signal was observed, because the reagent accumulated around the edge of the test zone where it was not contributing to the total colorimetric signal. When the reagent was deposited in the test zone, an even weaker colorimetric signal was observed because the reagent was pushed toward the edge of the test zone [22], and, in the case of the reagent pencil, a strong background signal was obtained due to the presence of the graphite in the pencil. These results have important implications for the design of a device. When performing colorimetric assays where all the components are water soluble, then the strongest signal will likely be obtained when the reagents are deposited in the sample zone. However, when preforming a capture assay, such as a lateral-flow immunoassay, then the reagents should be deposited in the channel of the device since the reagents will be captured at a test line and will not wick to the edge of a test zone.

**Figure 6:**
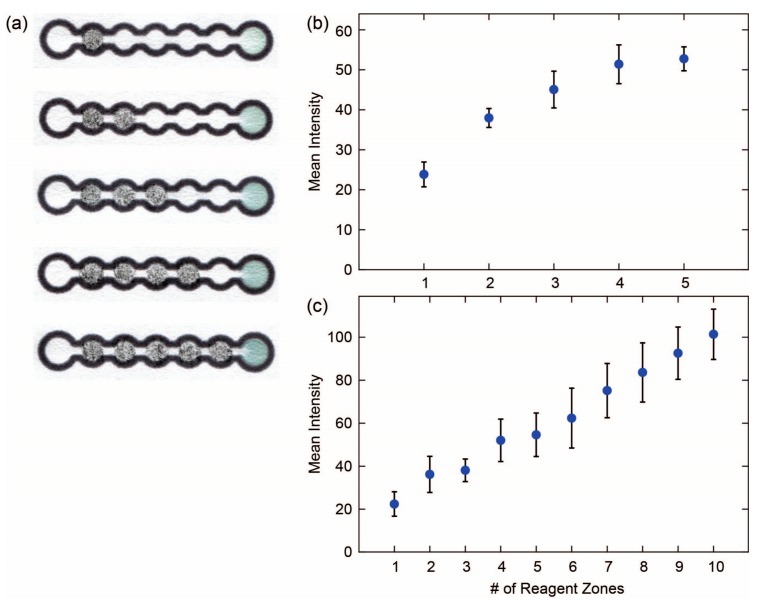
Reagent pencils for preparing calibration curves: (**a**) Photographs of devices after performing a glucose assay where the glucose was deposited using a reagent pencil in reagent zones on the device; (**b**) Plot of the mean intensity of the color in the test zone versus the number or reagent zones that were filled in with the pencil containing 5% *w*/*w* glucose. Data points represent the mean of five replicates and errors bar represent one standard deviation from the mean; (**c**) Plot of the mean intensity of the color in the test zone versus the number of reagent zones that were filled in with a pencil containing 5% *w*/*w* Erioglaucine. Data points represent the mean of five replicates and errors bar represent one standard deviation from the mean.

An additional advantage of depositing reagents in the channel of a device is that the amount of reagent deposited can be controlled by simply changing the surface area of the channel that is covered with the pencil trace. To provide the user with greater control over this process, we designed a caterpillar device with five reagent deposition zones and demonstrated that increasing amounts of reagent can be deposited on the device in a controlled manner by filling in more of the reagent deposition zones. Since both the front and back faces of the zones can be filled with pencil traces, it is possible to deposit up to 10 increments of reagent on this device. Using this approach, we were able to prepare a calibration curve for a glucose assay by filling increasing numbers of the reagent deposition zones (Figure 6a,b). The signal from the glucose assay became saturated after the addition of glucose to five zones, so we did not add reagent to the back face of the device. In the case of Erioglaucine, we saw an increase in the signal through the tenth reagent deposition zone. It should be possible to design devices with different numbers or different sizes of reagent deposition zones in order to provide additional control over the preparation of calibration curves using this method. The potential application of this method is in preparing calibration curves at the point of care for quantifying the results of a diagnostic assay without having to prepare a series of standard solutions of the reagent.

## 4. Conclusions

By characterizing reagent pencils in more detail, we can conclude that reagent pencils can be prepared for a wide variety of reagents using PEGs with molecular weights in the range of 2000–6000 g/mol. While the molecular weight of the PEG, concentration of reagent, and characteristics of the reagent all affected the wear of the pencil core, what ultimately influenced the amount of reagent released into the test zone most was the concentration of reagent in the pencil core and the location where the pencil was deposited on the device. Therefore, it should be possible to prepare reagent pencils for a variety of reagents using a variety of PEGs with relatively small differences between the pencils in terms of the amount of reagent deposited on the device and released into the test zone.

## Appendix A

**Table A1:** Complete results from the efficiency of reagent delivery experiments. Values represent the percentage of the total reagent deposited on the device that was recovered from each location on the device. Values are given as the mean ± standard deviation (*N* = 7).

Location	Reagent Pencil			Solution			
	Sample Zone	Channel	Test Zone	Sample Zone	Channel	Test Zone	Recovery
Sample zone	70 ± 5%	15 ± 3%	15 ± 4%	59 ± 4%	23 ± 3%	19 ± 5%	106 ± 11%
Channel	-	51 ± 7%	49 ± 7%	-	42 ± 3%	58 ± 3%	97 ± 6%
Test zone	-	-	100%	-	-	100%	102 ± 4%
